# Biopolymer Casein–Pullulan Coating of Fe_3_O_4_ Nanocomposites for Xanthohumol Encapsulation and Delivery

**DOI:** 10.3390/polym18020256

**Published:** 2026-01-17

**Authors:** Nikolay Zahariev, Dimitar Penkov, Radka Boyuklieva, Plamen Simeonov, Paolina Lukova, Raina Ardasheva, Plamen Katsarov

**Affiliations:** 1 Department of Pharmaceutical Technology and Biopharmacy, Faculty of Pharmacy, Medical University of Plovdiv, 4002 Plovdiv, Bulgaria; nikolay.zahariev@mu-plovdiv.bg (N.Z.); dimitar.penkov@mu-plovdiv.bg (D.P.); radka.boyuklieva@mu-plovdiv.bg (R.B.); plamen.simeonov@mu-plovdiv.bg (P.S.); 2 Research Institute at Medical University of Plovdiv, 4002 Plovdiv, Bulgaria; 3 Department of Pharmacognosy and Pharmaceutical Chemistry, Faculty of Pharmacy, Medical University-Plovdiv, Vasil Aprilov Str. 15A, 4002 Plovdiv, Bulgaria; paolina.lukova@mu-plovdiv.bg; 4 Department of Physics and Biophysics, Faculty of Pharmacy, Medical University-Plovdiv, Vasil Aprilov Str. 15A, 4002 Plovdiv, Bulgaria; rayna.ardasheva@mu-plovdiv.bg

**Keywords:** magnetic nanoparticles, casein–pullulan nanocomposites, xanthohumol delivery, theranostic platforms

## Abstract

**Introduction:** Magnetic nanoparticles are widely investigated as multifunctional platforms for drug delivery and theranostic applications, yet their biomedical implementation is hindered by aggregation, limited colloidal stability, and insufficient biocompatibility. Hybrid biopolymer coatings can mitigate these issues while supporting drug incorporation. **Aim:** This study aimed to develop casein–pullulan-coated Fe_3_O_4_ nanocomposites loaded with xanthohumol, enhancing stability and enabling controlled release for potential theranostic use. **Methods:** Fe_3_O_4_ nanoparticles were synthesized through co-precipitation and incorporated into a casein–pullulan matrix formed via polymer complexation and glutaraldehyde crosslinking. A 3^2^ full factorial design evaluated the influence of casein:pullulan ratio and crosslinker concentration on physicochemical performance. Nanocomposites were characterized for size, zeta potential, morphology, composition, and stability, while drug loading, encapsulation efficiency, and release profiles were determined spectrophotometrically. Molecular docking was performed to examine casein–pullulan interactions. **Results:** Uncoated Fe_3_O_4_ nanoparticles aggregated extensively, displaying mean sizes of ~292 nm, zeta potential of +80.95 mV and high polydispersity (PDI above 0.2). Incorporation into the biopolymer matrix improved colloidal stability, yielding particles of ~185 nm with zeta potentials near –35 mV. TEM and SEM confirmed spherical morphology and uniform magnetic core incorporation. The optimal formulation, consisting of a 1:1 casein:pullulan ratio with 1% glutaraldehyde, achieved 5.7% drug loading, 68% encapsulation efficiency, and sustained release of xanthohumol up to 84% over 120 h, fitting Fickian diffusion (Korsmeyer–Peppas R^2^ = 0.9877, n = 0.43). **Conclusions:** Casein–pullulan hybrid coatings significantly enhance Fe_3_O_4_ nanoparticle stability and enable controlled release of xanthohumol, presenting a promising platform for future targeted drug delivery and theranostic applications.

## 1. Introduction

In recent decades, nanotechnology has become a transformative tool in modern medicine and pharmacy, offering innovative strategies for diagnostics, therapy, and drug delivery. Among the various nanomaterials, magnetic nanoparticles (MNPs) have received particular attention due to their unique properties, small size, and adaptive surface chemistry. These features allow their use in both therapeutic and diagnostic applications, making them ideal candidates for the so-called “theranostics” [1].

MNPs are commonly based on iron oxide, such as magnetite (Fe_3_O_4_) or maghemite (γ-Fe_2_O_3_), although other magnetic materials have also been investigated (e.g., cobalt, nickel, and others). The ability to control their movement and localization by external magnetic fields distinguishes them from other nanosystems. This property, combined with versatile surface modification, makes them promising drug carriers, imaging agents, and therapeutic enhancers in various biomedical applications [2,3]. Several of their characteristics are essential for the biomedical potential of the MNPs, like: superparamagnetism—at sufficiently small size, MNPs exhibit superparamagnetism, meaning they show strong magnetization under an external magnetic field, but no residual magnetization once the field is removed, which prevents aggregation in vivo [4]; high surface area to volume ratio—nanoscale size enables high drug loading; surface functionalization—coating with different polymers (like PEG, chitosan, casein, dextran, lipids, and others) enhances colloidal stability, biocompatibility, and targeted delivery [5]. Probably their most important characteristic is their potential multifunctionality—the same MNP can integrate several roles such as drug delivery, imaging, and hyperthermia therapy in a single platform. Tissue engineering and regenerative medicine are other fields in which they can be beneficial. MNPs incorporated into scaffolds can influence cell growth and differentiation under magnetic stimulation. This approach has potential in bone repair, neural regeneration, and cardiac tissue engineering [6].

Despite their numerous advantages, MNPs still present several limitations that hinder their clinical translation. A significant challenge is colloidal instability arising from magnetic dipole–dipole interactions, which promotes aggregation and consequently affects biodistribution, circulation time, and drug delivery efficiency. To overcome this, surface functionalization is typically required to provide steric and electrostatic stabilization [3]. Another critical issue involves achieving controlled and predictable drug release, particularly in response to physiological or pathological stimuli. Concerns related to biocompatibility, toxicity, biodistribution, and clearance also remain. Although coated iron oxide nanoparticles are generally regarded as safe, unmodified magnetic cores can induce reactive oxygen species (ROS) generation and oxidative stress [2], while non-iron-based nanoparticles (e.g., nickel and cobalt) pose even greater cytotoxicity risks, necessitating careful surface engineering and dose regulation. Furthermore, MNPs are prone to rapid sequestration by the reticuloendothelial system, primarily in the liver and spleen, which may be advantageous for specific applications but reduces systemic circulation and limits targeted delivery to tumors or peripheral tissues. Long-term accumulation and biodegradation pathways are not yet fully understood and remain essential considerations. Additionally, achieving large-scale, reproducible synthesis with consistent particle size, magnetic behavior, and surface chemistry is technically demanding, and batch-to-batch variability continues to impede clinical applicability [7].

One of the most essential strategies to improve the biomedical performance of MNPs is surface coating. Non-coated iron oxide nanoparticles often suffer from aggregation, oxidation, and limited biocompatibility. Coating not only stabilizes the particles but also provides opportunities for drug loading, targeting, and controlled release. Among the most widely explored coatings are polysaccharides and proteins, due to their natural origin, biocompatibility, and functional versatility. Polysaccharides such as pullulan, dextran, chitosan, alginate, and others are commonly used to enhance the stability and functionality of MNPs. They provide steric hindrance and hydrophilicity, preventing aggregation in fluids and also reducing immune recognition and cytotoxicity. Different functional groups, such as carboxyl, hydroxyl, and amine, facilitate the conjugation of drugs, ligands, or targeting molecules. Pullulan, a natural water-soluble polysaccharide produced by Aureobasidium pullulans, has emerged as an excellent coating material due to its physicochemical and biological properties. Structurally composed of maltotriose units linked through α–(1-6) glycosidic bonds, pullulan exhibits high flexibility, hydrophilicity, and ease of chemical modification. Its biocompatibility, non-immunogenicity, and non-cytotoxicity make it particularly suitable. The hydrophilic nature of pullulan enhances the colloidal stability of MNPs in aqueous media, preventing aggregation and improving the dispersion and circulation in physiological environments. Additionally, its biodegradation into glucose units and its ability to form a “stealth” hydrophilic corona contribute to its long systemic circulation and low clearance by the reticuloendothelial system [8,9,10].

Proteins such as albumin, casein, and transferrin are also used as functional coatings for MNPs, offering biocompatibility, bioconjugation sites, stability, and reduced toxicity. Proteins mimic natural biological interfaces, decreasing immune clearance. Ligand-specific proteins can direct nanoparticles to particular receptors or inflamed tissues, improving therapeutic accuracy and enabling targeted delivery. Protein coatings can also be degraded by specific enzymes in diseased tissues, enabling site-specific drug release. Casein is a phosphoprotein naturally present in milk. It is amphiphilic and self-assembles into micellar structures, which allows it to encapsulate hydrophobic cores (such as Fe_3_O_4_ nanoparticles). Casein contains many functional groups (like -COOH, NH2, OH, phosphate), making it especially suitable for various chemical modifications and cross-linking [11,12].

The combination of polysaccharide and protein coatings can provide a synergistic strategy—polysaccharides ensure stability and functionalization, while proteins enhance targeting and biocompatibility. Hybrid coatings can also be tailored to respond to multiple stimuli (e.g., pH, enzymes, magnetic fields), thereby offering fine-tuned drug-delivery systems. In this context, hybrid systems integrating amphiphilic proteins with neutral polysaccharides are particularly attractive, as they combine efficient encapsulation of hydrophobic therapeutics with enhanced colloidal stability and controlled release behavior. Such designs offer a promising alternative to single-component biopolymer coatings, which often face limitations in drug loading efficiency or physiological compatibility.

Xanthohumol (XN) is a prenylated flavonoid derived from hops (Humulus lupulus) and is widely studied for its broad therapeutic potential, including antioxidant, anticancer, anti-inflammatory, antiviral, and neuroprotective activities [13,14]. Despite these promising biological effects, its clinical translation is significantly hindered by poor water solubility, low chemical stability, and extensive first-pass metabolism, resulting in low systemic bioavailability and limited therapeutic efficacy. Various formulation strategies have been developed to overcome these barriers. One common approach is the formation of inclusion complexes with cyclodextrins, which encapsulate hydrophobic molecules within their nonpolar cavity, thereby improving aqueous solubility, dissolution rate, and bioavailability [15,16]. However, while cyclodextrin complexes enhance solubility, they do not provide controlled release, protection against degradation, or targeted delivery. In contrast, encapsulation in nanoparticle-based systems can protect Xanthohumol from environmental degradation, improve dispersibility in aqueous media, enhance cellular internalization, and enable sustained release, making nanoparticle carriers particularly attractive for biomedical applications.

The current study aimed to develop a hybrid casein–pullulan-coated nanocomposite system loaded with Xanthohumol, featuring controlled release and potential theranostic applications.

## 2. Materials and Methods

Iron (II) sulfate heptahydrate, Iron (III) sulfate hydrate, Ammonia solution 25%, Glutaraldehide 25% (Mw 100.12 g/mol), Sodium Caseinate from bovine milk (Sigma-Aldrich, St. Louis, MO, USA), Xanthohumol (Mw 354.39 g/mol, Cayman chemicals, Ann Arbor, MI, USA), Pullulan (Mw 1.5 × 10^4^ g/mol, Tokyo Chemical Industry Co., Ltd., Tokyo, Japan), Hydroxypropyl-β-cyclodextrin (Mw ~ 1460, Sigma-Aldrich, St. Louis, MO, USA). All other reagents were of analytical grade.

### 2.1. Preparation of Fe_3_O_4_ Magnetic Nanoparticles

The Fe_3_O_4_ MNPs were prepared by co-precipitation following the procedure reported by Kresnik et al. [17], with minor modifications. Briefly, 0.37 g of iron (II) sulfate heptahydrate and 0.29 g of iron (III) sulfate hydrate were dissolved in 100 mL of distilled water under continuous stirring to obtain a clear solution (pH 3). After complete dissolution of the salts, 15 mL of 2% ammonia solution was added dropwise under stirring. The pH was maintained at 3 for 30 min, after which 25 mL of concentrated ammonia solution (25%) was added, and the mixture was stirred for an additional 30 min. Upon nanoparticle formation, a magnet was placed at the bottom of the beaker, and the nanosuspension was allowed to sediment. The supernatant was carefully decanted, and the Fe_3_O_4_ MNPs were rinsed several times with distilled water.

### 2.2. Preparation of Casein–Pullulan-Coated Fe_3_O_4_ Magnetic Nanocomposites

Casein–pullulan-coated (Cas/Pull) Fe_3_O_4_ magnetic nanocomposites were prepared via polymer complexation. Fixed volumes of casein (15 mL) and pullulan (15 mL) aqueous solutions were used, while their concentrations were varied according to the 3^2^ factorial design to obtain the required casein:pullulan mass ratios (2:1, 1:1, 1:2). Initially, 2 mL of a 0.06% (*w*/*v*) Fe_3_O_4_ suspension (0.6 mg/mL) were added to the casein solution (pH 7) and stirred for 30 min. The mixture was then added dropwise to the pullulan solution under continuous stirring at 3000 rpm (Witeg HS-100D, Witeg Labortechnik GmbH, Wertheim, Germany), enabling formation of the casein–pullulan complex. Glutaraldehyde working solutions (0.5%, 1.0%, 1.5% *v*/*v*) were prepared by diluting the 25% stock. Then, 200 μL of the respective glutaraldehyde solution were added to crosslink and stabilize the network. The final nanosuspension was lyophilized for 24 h at −55 °C and 0.1 mbar (Alfa 1–2 LSCbasic, Martin Christ, Osterode am Harz, Germany).

### 2.3. Preparation of Casein–Pullulan-Coated Fe_3_O_4_ Magnetic Nanocomposites Loaded with Xanthohumol

Casein–pullulan-coated Fe_3_O_4_ magnetic nanocomposites loaded with XN were prepared using the same complexation and crosslinking strategy described above, with the additional incorporation of an XN inclusion complex.

#### 2.3.1. Preparation of Hydroxypropyl-β-Cyclodextrin/Xanthohumol

The hydroxypropyl-β-cyclodextrin/Xanthohumol (HP-β-CD/XN) inclusion complex was obtained via the solvent evaporation method [18] at a 1:1 (*w*/*w*) ratio. HP-β-CD (10 mg) was dissolved in 10 mL of distilled water, while XN (10 mg) was dissolved in 2 mL of methanol. The XN solution was added dropwise to the cyclodextrin solution under continuous stirring (500 rpm), and the mixture was stirred for 1 h to evaporate the methanol completely.

#### 2.3.2. Incorporation of Hydroxypropyl-β-Cyclodextrin/Xanthohumolcomplex into Casein–Pullulan Fe_3_O_4_ Nanocomposites

The HP-β-CD/XN complex (10 mL) was added to the Fe_3_O_4_–casein dispersion prepared as described in Section 2.2, and stirred for 15 min. The mixture was then introduced into the pullulan solution (fixed volume, variable concentration) under stirring (3000 rpm). Crosslinking was performed by adding 200 μL of glutaraldehyde at 0.5%, 1.0%, or 1.5% *v*/*v*, as specified in the experimental design. The final nanosuspension was lyophilized for 24 h at −55 °C and 0.1 mbar.

#### 2.3.3. Experimental Design Parameters

A 3^2^ full factorial experimental design was employed to investigate the influence of formulation parameters on the characteristics of XN-loaded casein–pullulan Fe_3_O_4_ magnetic nanocomposites. The independent variables were the casein:pullulan mass ratio (X_1_) and the glutaraldehyde concentration (X_2_), each studied at three levels (−1, 0, +1) (Table 1), resulting in nine experimental formulations (Table 2). The dependent variables were particle size (Y_1_), zeta potential (Y_2_), drug loading (DL, Y_3_), and encapsulation efficiency (EE, Y_4_). Each batch contained 1.2 mg Fe_3_O_4_ (from 2 mL of 0.06% suspension) and 10 mg XN.

Statistical analysis was performed using Minitab 21.2 software (Minitab LLC, State College, PA, USA). The significance of the regression models and the individual terms was evaluated using one-way analysis of variance (ANOVA) at a 95% confidence level (*p* < 0.05).

### 2.4. Characterization

#### 2.4.1. Production Yield

The production yields of the composite nanomaterials from different batches were calculated using the weight of the obtained nanocomposites with respect to the initial quantity of the used drug, Fe_3_O_4,_ and biopolymers, according to the following equation:

(1) $$Production\;Yield\left(\%\right)=\frac{Obtained\;nanoparticles\;(mg)}{XN\left(mg\right)+CD\;(mg)+Casein\left(mg\right)+Pullulan\left(mg\right)+MNPs\;(mg)}\times 100$$

#### 2.4.2. Particle Size, Size Distribution, and Zeta Potential

The particle sizes of the Fe_3_O_4_ nanoparticles, blank Cas/Pull–Fe_3_O_4_ nanocomposites, and XN-loaded Cas/Pull–Fe_3_O_4_ nanocomposites were analyzed using dynamic light scattering (DLS) (Zetasizer Ultra Red, Malvern Panalytical, Malvern, UK). The device was fitted with a 10 mW, 632.8 nm laser, and measurements were carried out using Non-Invasive Backscatter (NIBS) at 173°. A specific amount of particles was dispersed in distilled water and sonicated in a bath sonicator (Sonorex Bandelin electronic, Berlin, Germany) for 5 min. The samples were subsequently diluted 1:30 with distilled water to minimize multiple scattering.

#### 2.4.3. Physical Stability

A predetermined amount of particles from each batch was ultrasonically dispersed in phosphate-buffered saline (PBS, pH 7.4), and changes in particle size and zeta potential over one week were evaluated using dynamic light scattering (Zetasizer Ultra Red, Malvern Panalytical, Malvern, UK).

#### 2.4.4. Drug Loading and Encapsulation Efficiency

Drug loading (DL) and encapsulation efficiency (EE) were determined spectrophotometrically. Twenty milligrams of XN-loaded nanocomposites were dispersed in 10 mL of a 1:1 (*v*/*v*) mixture of distilled water and methanol and incubated for 24 h to extract XN. Afterward, 1 mL of the extract was diluted with 10 mL of distilled water and filtered through a 0.22 µm Chromafil^®^ filter (Macherey-Nagel, Düren, Germany). XN concentration was determined using a UV–Vis spectrophotometer (Evolution 3000 Pro, Thermo Scientific, Waltham, MA, USA) at 369 nm. DL and EE were calculated as follows:

(2) $$\mathrm{DL}\;(\%)\;=\frac{Amount\;of\;drug\;in\;the\;formulation\;(mg)}{Total\;amount\;of\;nanoparticles\;(mg)}\times 100$$

(3) $$\mathrm{EE}\;(\%)=\frac{Amount\;of\;drug\;in\;the\;formulation\;(mg)}{Theoretical\;drug\;content\;(mg)}\times 100$$

#### 2.4.5. Scanning Electron Microscopy and Energy-Dispersive X-Ray Spectroscopy (SEM/EDX)

The morphology and elemental composition of blank and XN-loaded Cas/Pull–Fe_3_O_4_ nanocomposites were examined using SEM (Prisma E, Thermo Scientific, USA) equipped with an EDX detector (Thermo Scientific, USA). To evaluate the elemental composition and verify the successful incorporation of Fe_3_O_4_ MNPs within the casein–pullulan matrix, EDX spectra were recorded using the Point ID method across 10 randomly selected areas throughout the sample. The spectra were recorded at an accelerating voltage of 20 kV and a working distance of 10 mm. The method was used to determine the coexistence of inorganic core (Fe) and the main organic shell components (C,O).

#### 2.4.6. High-Resolution Transmission Electron Microscopy (HRTEM)

The structural and morphological characteristics of the developed nanoparticles were determined by transmission electron microscopy (TEM) (Talos F200C Thermo Fisher Scientific, Waltham, MA, USA). The conducted selected area electron diffraction (SAED) analysis provided molecular-scale insight into the crystalline phase of the synthesized bare Fe_3_O_4_ nanoparticles and the incorporated within the polymer matrix. Suspensions of Fe_3_O_4_ nanoparticles, blank nanocomposites, and XN-loaded nanocomposites were drop-cast on 200-mesh formvar/carbon-coated copper grids and imaged at 200 kV.

#### 2.4.7. Molecular Docking

To elucidate the interactions between casein and pullulan, molecular docking was performed using AutoDock Vina 1.2.0. The casein structure (chain P, PDB ID: 7TTS) was downloaded from the Protein Data Bank (https://www.rcsb.org), and all water molecules and unwanted structures were removed. The ligand pullulan was obtained from Thermo Fisher Scientific in SMILES format [19,20]. A blind docking approach was applied using a 100 × 100 × 100 Å grid with a 0.5 Å spacing, followed by refinement using a 20 × 20 × 20 Å grid centered on the predicted binding region.

#### 2.4.8. In Vitro Release of Xanthohumol

XN release was studied using the dialysis bag method. A 12 kDa MWCO dialysis membrane (Sigma, MWCO 12 kDa) was hydrated in distilled water for 24 h. Nanoparticles equivalent to 0.750 mg XN were dispersed in 5 mL of dissolution media (PBS 7.4) and then placed in a dialysis bag sealed with a plastic clamp. Each bag was then immersed in a beaker containing 20 mL of PBS 7.4 and maintained under constant gentle agitation on an electromagnetic stirrer at 150 rpm and 37.0 ± 0.5 °C. Aliquots of 2 mL were withdrawn at set intervals and replaced with fresh medium. Samples were filtered using 0.45 μm Chromafil^®^ membranes and analyzed by UV–Vis spectrophotometry (Thermo Scientific, Waltham, MA, USA) at 369 nm.

## 3. Results and Discussion

### 3.1. Physicochemical Characterization of Bare Fe_3_O_4_ Magnetic Nanoparticles

**Figure 1:**
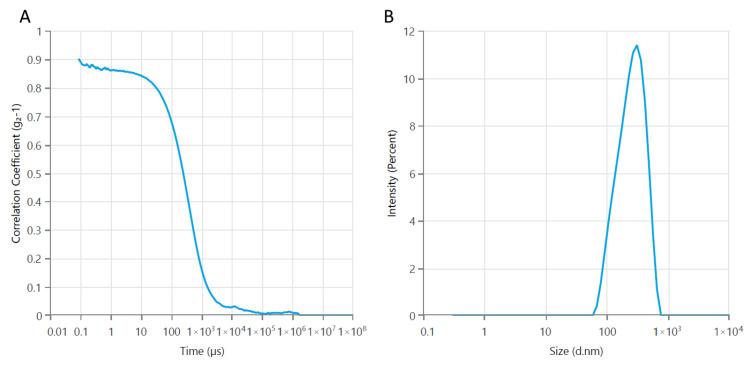
Average particle size of Fe_3_O_4_ magnetic nanoparticles: (**A**) a correlogram; (**B**) size distribution by intensity.

DLS analysis showed that the synthesized Fe_3_O_4_ nanoparticles exhibited an average hydrodynamic diameter of 292 nm, a PDI of 0.44, and a high positive zeta potential of +80.95 mV (Figure 1). The elevated PDI indicates a broad size distribution, likely resulting from agglomeration of the primary nanoparticles [20,21].

**Figure 2:**
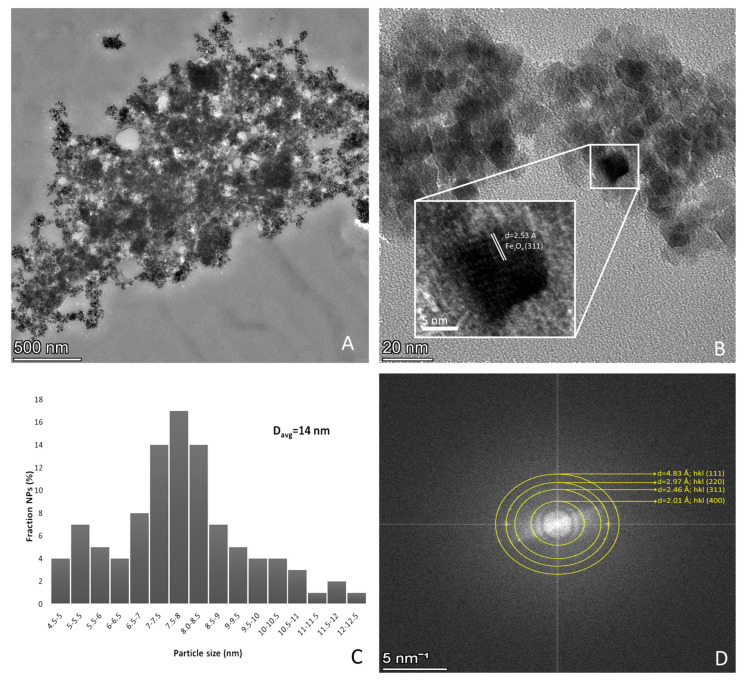
Fe_3_O_4_ magnetite nanoparticles: (**A**) TEM image; (**B**) interplanar spacing evaluation; (**C**) average particle size and particle size distribution, based on TEM analysis; (**D**) selected area electron diffraction analysis.

TEM imaging confirmed this assumption, revealing densely packed aggregates with micrometer-scale dimensions (Figure 2). Measurement of 100 individual particles within the agglomerates demonstrated that the average particle size of the developed nanoparticles is 14 ± 4 nm (Figure 2C). The conducted selected area electron diffraction (SAED) confirmed the successful formation of magnetite phase. The analysis determined interplanar spacings (d-spacings) derived from the diffraction rings were d = 4.83 Å, d = 2.97 Å, d = 2.48Å, and d = 2.01 Å (Figure 2D) correspond precisely to the (1 1 1), (2 2 0), (3 1 1), and (4 0 0) crystallographic planes (JCPDS No. 19-0629), demonstrating the successful formation of a high-purity magnetite (Fe_3_O_4_) nanoparticles. Figure 2B shows the HRTEM image of a single Fe_3_O_4_ nanoparticle, as the obtained lattice pattern demonstrated that this particle was a single perfect cubic crystal of Fe_3_O_4_ with a d-spacing of 2.53 Å at the (3 1 1) reflection [22].

### 3.2. Physicochemical Characterization of Casein–Pullulan-Coated Fe_3_O_4_ Nanocomposites

To improve the colloidal stability of Fe_3_O_4_ MNPs and mitigate their inherent tendency to aggregate, the particles were incorporated into a casein–pullulan polymeric matrix. Casein, an amphiphilic phosphoprotein, interacts with magnetite via electrostatic attraction and coordination of Fe^3+^ ions by phosphate groups, whereas pullulan, a biocompatible non-allergenic polysaccharide, enhances stability and structural integrity of the composite [23]. The following analyses were conducted to evaluate the physicochemical properties of the obtained nanocomposites.

#### 3.2.1. Particle Size and Zeta Potential

**Figure 3:**
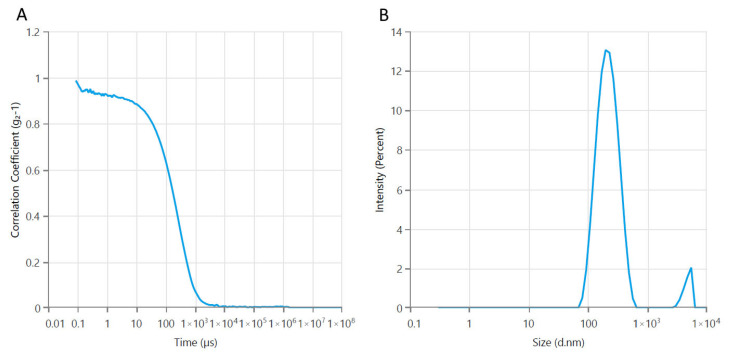
Average particle size of the developed composite structures: (**A**) a correlogram; (**B**) size distribution by intensity.

DLS analysis revealed that the incorporation of Fe_3_O_4_ nanoparticles into the casein–pullulan complex resulted in composite structures with an average hydrodynamic diameter of 185 nm and a zeta potential of −34.71 mV. The increase in particle size compared to bare magnetite nanoparticles can be attributed to the formation of a continuous biopolymer coating around the Fe_3_O_4_ cores. The negative surface charge reflects the presence of deprotonated carboxyl and phosphate groups from casein and hydroxyl groups from pullulan, contributing to improved colloidal stability (Figure 3).

#### 3.2.2. High-Resolution TEM Analysis

High-resolution TEM analysis confirmed that the composite nanostructures exhibited a predominantly spherical morphology, with an average particle diameter of approximately 200 nm and a coating layer of around 13 nm. After incorporation into the casein–pullulan composite matrix, the diffraction patterns retained their characteristic ring structure with negligible deviations in d-spacing, such as the (3 1 1) plane at 2.47 Å, (2 2 0) plane at 2.97 Å and (1 1 1) plane at 4.84 Å. This stability indicates that the magnetite nanoparticles maintained their structural integrity and were effectively encapsulated without undergoing phase transformation or oxidation.

**Figure 4:**
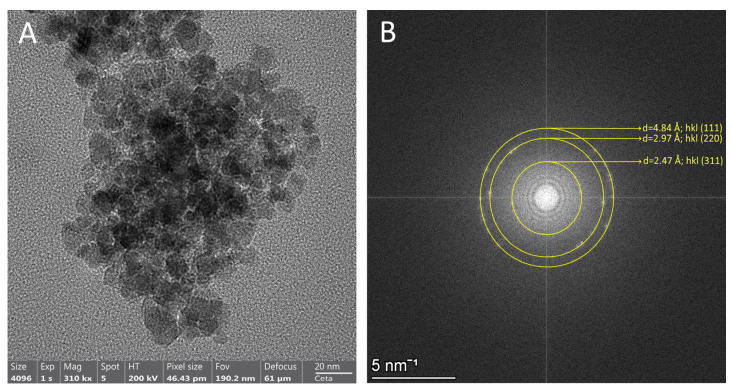
Fe_3_O_4_ magnetite nanoparticles included in casein–pullulan complex: (**A**) TEM image; (**B**) selected area electron diffraction analysis.

Furthermore as shown in Figure 4, the incorporation of Fe_3_O_4_ nanoparticles into the casein–pullulan matrix significantly reduced particle agglomeration, resulting in more discrete particles and improved colloidal stability [24].

#### 3.2.3. SEM-EDX Elemental Analysis of the Composite

SEM–EDS analysis was performed to assess the degree of magnetite incorporation within the polymeric matrix. Three batches were prepared using a fixed Fe_3_O_4_ content (2 mL of 0.06% suspension), a constant 1:1 casein:pullulan ratio, and different glutaraldehyde concentrations (0.5%, 1.0%, 1.5%).

All analyzed samples exhibited detectable iron signals, confirming successful incorporation of Fe_3_O_4_ nanoparticles into the composite matrix. The average Fe content ranged from 0.26% to 0.7%, depending on the crosslinker concentration (Figure S2). An increase in glutaraldehyde concentration resulted in higher Fe incorporation, indicating that denser crosslinked networks promote more efficient immobilization of Fe_3_O_4_ nanoparticles within the polymeric structure (Table 3) [25].

#### 3.2.4. Colloidal Stability in PBS

**Figure 5:**
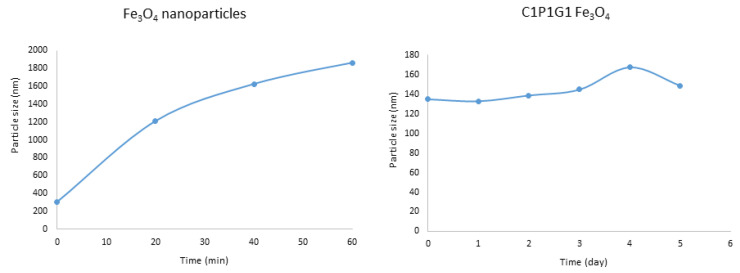
Colloidal physical stability of magnetic nanoparticles and magnetite nanoparticles incorporated in casein–pullulan complex (C1P1G1 Fe_3_O_4_).

To evaluate colloidal stability under physiological conditions, the samples were dispersed in PBS (pH 7.4), and changes in particle size were monitored over a period of five days. Bare Fe_3_O_4_ nanoparticles rapidly aggregated, with the average particle size increasing from 307 nm at the initial time point to approximately 1.6 µm within 30 min (Figure 5), primarily due to strong magnetic dipole–dipole interactions [26].

In contrast, casein–pullulan-coated Fe_3_O_4_ nanoparticles maintained stable hydrodynamic diameters in the range of 135–168 nm throughout the study (Figure 5). This pronounced stabilization effect can be attributed to the steric hindrance and surface charge provided by the polymeric coating. Similar enhancements in colloidal stability have been reported for polymer-functionalized magnetic nanoparticles [26].

Based on these observations, a molecular docking analysis was subsequently conducted to elucidate the molecular interactions underlying casein–pullulan complex formation and the experimentally observed improvement in colloidal stability.

#### 3.2.5. Molecular Docking Analysis

The molecular docking analysis provided structural insight into the interactions responsible for the formation and stability of the experimentally observed casein–pullulan complexes. The results indicate that the association between casein and pullulan is predominantly triggered by intermolecular hydrogen bonding, arising from the high density of hydroxyl (−OH) and amide (−NH) functional groups present in both biopolymers [27,28,29].

**Figure 6:**
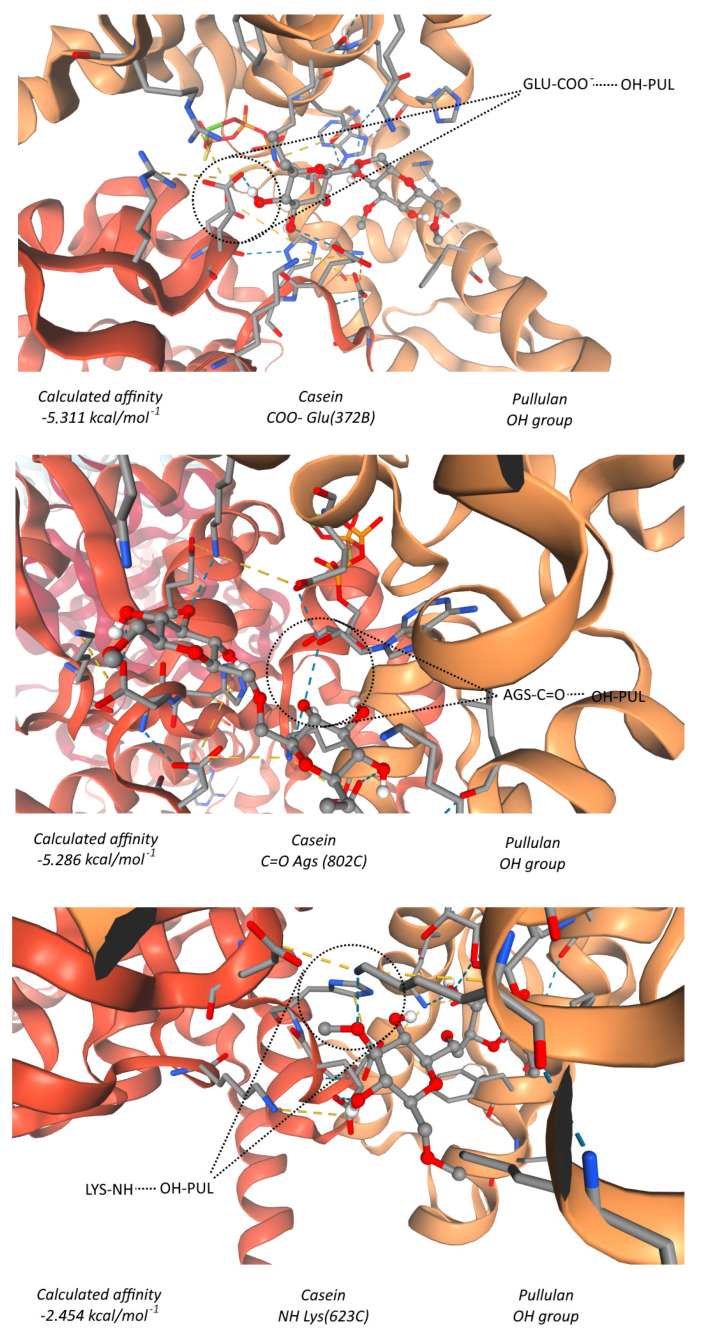
Graphical representation of the predicted bonds between beta-casein and pullulan and their calculated affinities.

Pullulan contains multiple hydroxyl groups along its α-(1→4) and α-(1→6) linked glucose residues, which can act as both hydrogen-bond donors and acceptors. Casein, rich in polar amino acid residues such as serine, threonine, glutamic acid, and aspartic acid, provides several hydrogen-bonding sites, including peptide carbonyl (C=O), amide (−NH), hydroxyl (−OH), and carboxylate (−COO^−^) groups [27,28]. At the molecular level, the dominant interactions observed in the docking simulations include hydrogen bonds formed between pullulan hydroxyl groups and carbonyl oxygens of the peptide backbone (−OH···O=C), between pullulan hydroxyl oxygens and amide protons of casein (−OH···NH), and between pullulan hydroxyl groups and deprotonated carboxylate groups (−COO^−^) within casein side chains (Figure 6) [27,28,30].

The calculated binding affinities for the most favorable interactions ranged from −2.45 to −5.31 kcal·mol^−1^, which corresponds to moderate to strong hydrogen bonds within the typical range reported for non-covalent biomolecular interactions [31]. Notably, interactions involving pullulan hydroxyl groups and carboxylate (Glu372B) or carbonyl groups (Ags802C) of casein exhibited binding energies around −5 kcal·mol^−1^, suggesting their dominant contribution to complex stabilization (Figure 6). These values fall within the established range for intermolecular hydrogen bonds (1–14 kcal/mol), confirming that hydrogen bonding is the principal driving force behind the casein–pullulan association. [31].

Overall, the docking results support the experimental findings by confirming that casein–pullulan complex formation is energetically favorable and primarily driven by a dense hydrogen-bonding network. These molecular-level interactions rationalize the improved colloidal stability and reduced aggregation observed for casein–pullulan-coated Fe_3_O_4_ nanoparticles.

### 3.3. Optimization of Xanthohumol-Loaded Casein–Pullulan Fe_3_O_4_ Nanocomposites via 3^2^ Factorial Design

Xanthohumol was selected as a hydrophobic model drug to evaluate the suitability of the casein–pullulan-coated Fe_3_O_4_ nanocomposites as a potential drug delivery system [12,13,14,15]. A 3^2^ full factorial design was applied (Section 2.3.3) to systematically investigate the influence of two formulation variables, namely the casein:pullulan mass ratio (X_1_) and the glutaraldehyde concentration (X_2_), on the physicochemical properties of the nanocomposites and their drug incorporation performance [32]. The studied responses included particle size (Y1), zeta potential (Y2), DL (Y3), and EE (Y4).

The main physicochemical characteristics of the obtained nanocomposites are summarized in Table 4. The results indicate a pronounced dependence of particle size and drug incorporation parameters on formulation composition, while all formulations exhibited negative zeta potentials, suggesting electrostatically stabilized colloidal systems.

The statistical significance of the main and interaction effects of the independent variables on the studied responses was evaluated by analysis of variance (ANOVA). Based on this statistical evaluation, the effects of the independent variables on particle size, zeta potential, and drug incorporation parameters are discussed in detail in the following sections.

Based on the obtained data from the ANOVA, in terms of F-value and *p*-values, the statistical significance of each of the independent variables, as well as their combined interaction on the investigated dependent variables, can be determined. The independent variables that demonstrate the highest F-values and *p* < 0.05 are the main factors with a statistically significant impact on the studied dependent variables

The results show that the linear increase in casein to pullulan ratio, glutaraldhyde concentration, as well as their interaction, have a statistically significant effect on the zeta potential (*p* < 0.05) and the EE (*p* < 0.05). Regarding the particle size only the casein-to-pullulan ratio (*p* < 0.05) and the interaction between the casein-to-pullulan ratio and glutaraldehyde concentration (*p* < 0.05) had a statistically significant effect. As for the drug loading, only the casein to fucoidan ratio (*p* < 0.05) and the glutaraldehyde concentration (*p* < 0.05) demonstrated statistically significant impact on the studied variable. The standardized effects of the independent variables are also represented by Pareto charts, which rank the effects of the independent variables on the dependent variables in terms of their significances [33].

#### 3.3.1. Effect of the Independent over Particle Size (Effect of Formulation Variables on Particle Size)

**Figure 7:**
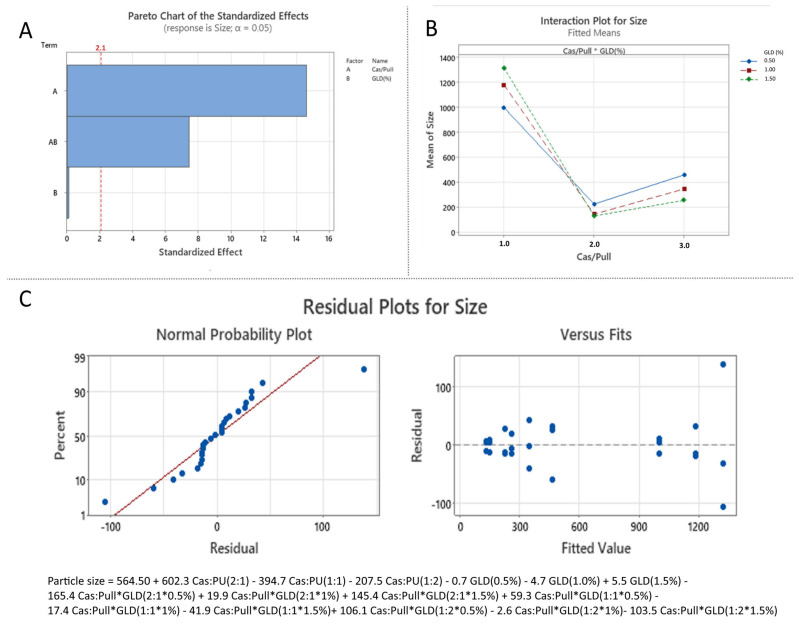
Statistical analysis of the effect of independent variables on particle size: (**A**) Pareto chart (A corresponds to X_1_, B corresponds to X_2_ and AB corresponds to X_1_X_2_); (**B**) Interaction plot (1.0 corresponds to Cas:Pull 2:1, 2.0 corresponds to Cas:Pull 1:1 and 3.0 corresponds to Cas:Pull 1:2); (**C**) Normal probability plot of residuals with respect to size and plot of residuals versus predicted size values.

The statistical significance of the formulation variables on particle size was presented via Pareto chart (Figure 7A), which confirms the results of the ANOVA analysis and demonstrates that the casein:pullulan mass ratio (X_1_) and its interaction with glutaraldehyde concentration (X_1_X_2_) exert a statistically significant effect on particle size (*p* < 0.05).

The influence of the independent variables and their interaction on particle size is further illustrated by the interaction plots (Figure 7B). The particle size of the nanocomposites varied broadly, ranging from 140 ± 8 nm to 1212 ± 102 nm (Table 4). The smallest particles (140 ± 8 nm to 256 ± 19 nm) were consistently obtained at a 1:1 casein:pullulan ratio, suggesting efficient polymer complexation and formation of compact nanostructures [33]. In contrast, increasing the casein content (2:1 ratio) led to a pronounced increase in particle size, likely due to inefficient polymer complexation. Similarly, increasing the pullulan amount (1:2 ratio) resulted in larger particles (278 ± 14 nm to 495 ± 42 nm), which can be attributed to the formation of a thicker polysaccharide coating [34].

The effect of glutaraldehyde concentration was dependent on polymer stoichiometry. At 1:1 and 1:2 casein:pullulan ratios, increasing the crosslinker concentration resulted in reduced particle size, consistent with the formation of denser, more compact polymer networks [35]. Conversely, at a 2:1 casein:pullulan ratio, increasing glutaraldehyde concentration led to particle enlargement, likely due to crosslinking between loosely associated polymer aggregates and the formation of larger interconnected structures [35].

The model exhibited excellent goodness-of-fit (R^2^ = 0.9917, R^2^adj = 0.9880), indicating that approximately 98% of the variability in particle size could be explained by the selected formulation parameters. The statistical significance and predictive capacity of the model were further confirmed by ANOVA (*p* < 0.05). An analysis of the residuals demonstrated normal distribution and random scatter around zero (Figure 7C), confirming the adequacy of the developed model for predicting particle size within the studied design space.

#### 3.3.2. Effect of Formulation Variables on Zeta Potential

**Figure 8:**
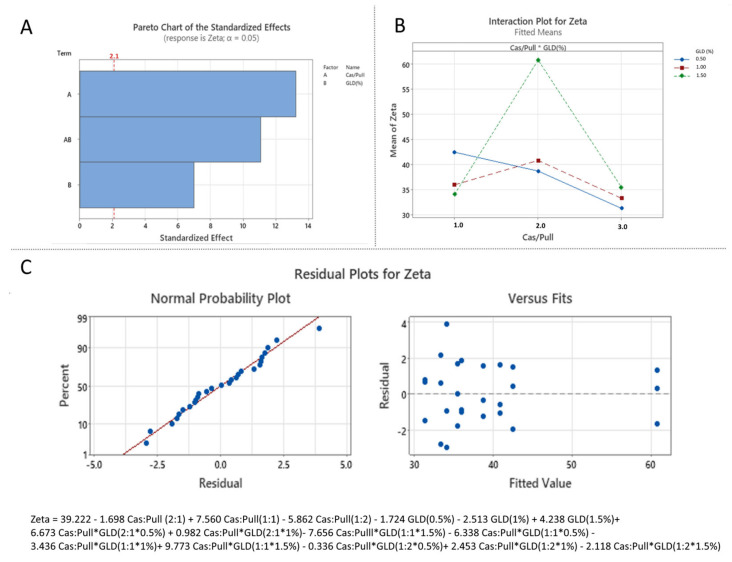
Statistical analysis of the effect of independent variables on zeta potential: (**A**) Pareto chart (A corresponds to X_1_, B corresponds to X_2_ and AB corresponds to X_1_X_2_); (**B**) Interaction plot (1.0 corresponds to Cas:Pull 2:1, 2.0 corresponds to Cas:Pull 1:1 and 3.0 corresponds to Cas:Pull 1:2); (**C**) Normal probability plot of residuals with respect to zeta potential and plot of residuals versus predicted zeta potential values.

The standardized effects of the formulation variables on the zeta potential were presented via Pareto chart (Figure 8A). The analysis confirms that the casein:pullulan ratio (X_1_), glutaraldehyde concentration (X_2_), and their interaction (X_1_X_2_) have a statistically significant influence on the surface charge of the nanocomposites (*p* < 0.05).

The combined effects of the independent variables on zeta potential are further illustrated by interaction plots (Figure 8B). Across all formulations, zeta potential values remained negative, ranging from −32.11 ± 1.1 mV to −59.14 ± 1.2 mV (Table 4), indicating electrostatic stabilization of the nanocomposite dispersions. Compared to the formulations prepared at a 1:1 casein:pullulan ratio, both asymmetric ratios (2:1 and 1:2) resulted in less negative zeta potential values [35]. Increasing the pullulan content (1:2 ratio) led to higher zeta potential values, which can be attributed to the dominance of neutral polysaccharide chains at the particle surface, as also supported by TEM [36]. In contrast, increasing the casein content (2:1 ratio) resulted in reduced absolute zeta potential values, likely due to less efficient polymer complexation and altered surface charge [36].

The effect of glutaraldehyde concentration was also dependent on polymer stoichiometry. In formulations prepared at 1:1 and 1:2 casein:pullulan ratios, increasing the glutaraldehyde concentration from 0.5% to 1.5% led to a gradual decrease in the absolute value of the zeta potential. This trend can be attributed to the reaction of glutaraldehyde with protonated amine groups (NH_3_^+^) in the casein structure, resulting in partial neutralization of surface charge [36,37,38]. Notably, the most negative zeta potential values (−40.31 ± 1.2 mV to −59.14 ± 1.2 mV), corresponding to enhanced colloidal stability, were observed for nanocomposites prepared at a 1:1 casein:pullulan ratio. An opposite trend was observed for formulations with a 2:1 casein:pullulan ratio, where increasing glutaraldehyde concentration led to higher zeta potential values, possibly due to the formation of intermolecular bridges between casein-rich domains [25].

The developed model demonstrated good predictive performance, with R^2^ = 0.9634 and R^2^adj = 0.9471, indicating that approximately 94% of the variance in zeta potential could be explained by the selected formulation parameters. The statistical significance and adequacy of the model were further confirmed by ANOVA (*p* < 0.05).

Residual analysis revealed normal distribution and random dispersion around zero (Figure 8C), confirming the reliability of the regression model and its suitability for predicting zeta potential within the investigated experimental domain.

#### 3.3.3. Effect of the Independent over DL and EE

**Figure 9:**
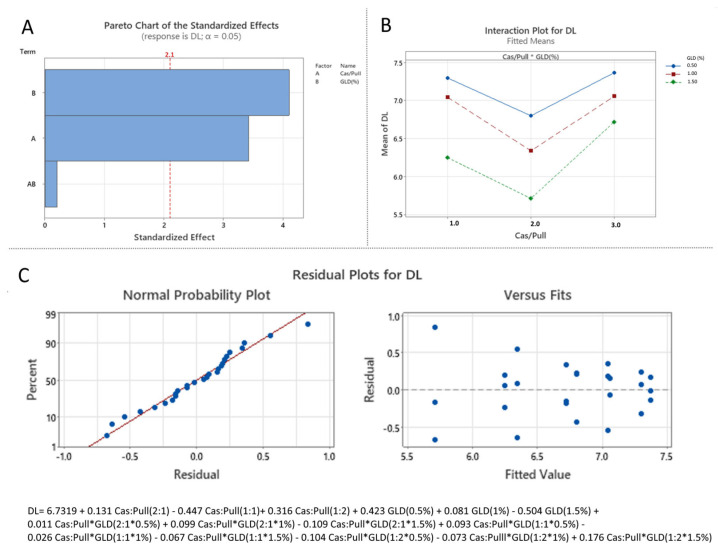
Statistical analysis of the effect of independent variables on drug loading: (**A**) Pareto chart (A corresponds to X_1_, B corresponds to X_2_ and AB corresponds to X_1_X_2_); (**B**) Interaction plot (1.0 corresponds to Cas:Pull 2:1, 2.0 corresponds to Cas:Pull 1:1 and 3.0 corresponds to Cas:Pull 1:2); (**C**) Normal probability plot of residuals with respect to drug loading and plot of residuals versus predicted drug loading values.

**Figure 10:**
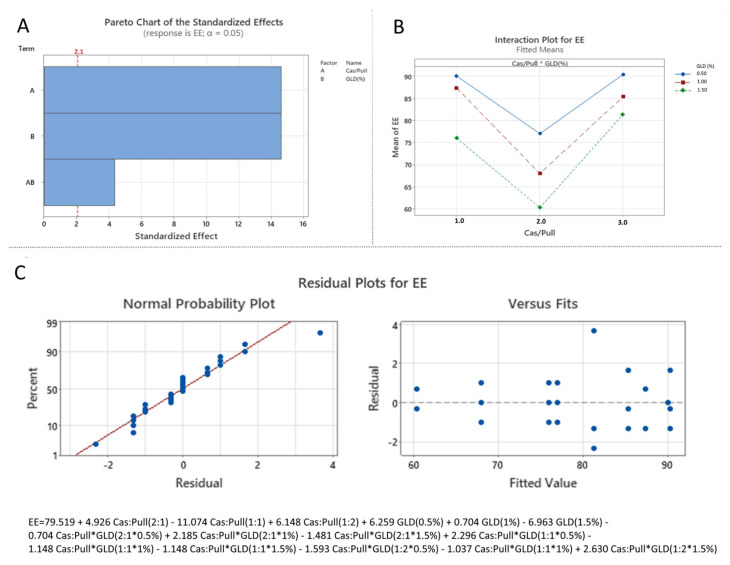
Statistical analysis of the effect of independent variables on encapsulation efficacy: (**A**) Pareto chart (A corresponds to X_1_, B corresponds to X_2_ and AB corresponds to X_1_X_2_); (**B**) Interaction plot (1.0 corresponds to Cas:Pull 2:1, 2.0 corresponds to Cas:Pull 1:1 and 3.0 corresponds to Cas:Pull 1:2); (**C**) Normal probability plot of residuals with respect to encapsulation efficacy and plot of residuals versus predicted encapsulation efficacy values.

The standardized effects of the formulation variables on DL and EE were presented via Pareto charts (Figure 9A and Figure 10A). The analysis indicates that both the casein:pullulan ratio (X_1_) and glutaraldehyde concentration (X_2_) exert a statistically significant effect on EE (*p* < 0.05). In contrast, DL was significantly influenced by the main effects of X_1_ and X_2_, while their interaction was not statistically significant (*p* > 0.05).

The combined influence of the formulation variables is further illustrated by interaction plots (Figure 9B and Figure 10B). DL values ranged from 5.4 ± 0.6% to 7.5 ± 0.2%, while EE varied from 68 ± 0.8% to 90 ± 0.9% (Table 4). Compared to formulations prepared at a 1:1 casein:pullulan ratio, nanocomposites obtained at asymmetric ratios (2:1 and 1:2) exhibited increased DL and EE. At a 1:2 ratio (pullulan-rich), the higher EE can be attributed to the formation of a thicker polymeric coating, facilitating incorporation of xanthohumol within both the core and shell of the nanocomposites [39,40]. Conversely, at a 2:1 ratio (casein-rich), the increased DL and EE are likely related to the formation of larger composite structures capable of entrapping higher drug amounts.

Increasing the glutaraldehyde concentration resulted in a gradual decrease in both DL and EE across all formulations, which can be attributed to the formation of denser crosslinked networks that limit drug accumulation within the polymer matrix [39,40]. The statistical significance of these effects was confirmed by one-way ANOVA.

The developed models showed satisfactory predictive performance (DL: R^2^ = 0.6912; EE: R^2^ = 0.9847), with residual analysis indicating acceptable normality and distribution (Figure 9C and Figure 10C).

After evaluating the particle size, zeta potential, DL, and EE, the formulation with a casein-to-pullulan mass ratio of 1:1, crosslinked with 1.0% glutaraldehyde (Fe_3_O_4_–C1P1G1–XN), was determined to be the optimal choice. This formulation demonstrated a favorable combination of nanoscale particle size, sufficiently high negative zeta potential, and effective drug incorporation, which are summarized in Table 4.

### 3.4. TEM Analysis of the Structure and Surface Morphology of the Developed Fe_3_O_4_ C1P1G1 XN Nanocomposites

**Figure 11:**
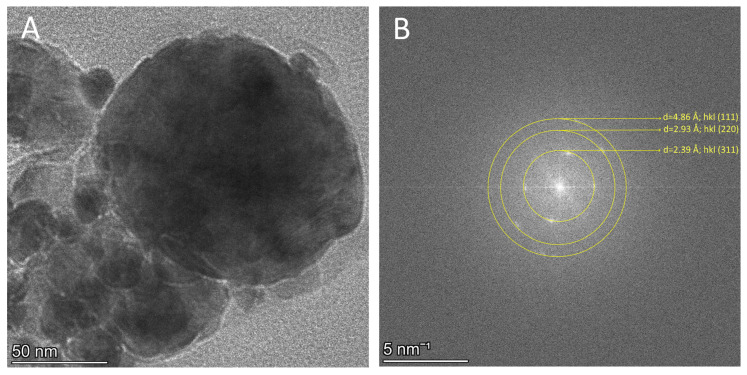
Fe_3_O_4_-C1P1G10-XN nanocomposites: (**A**) TEM image; (**B**) selected area electron diffraction analysis.

TEM analysis revealed that the selected Fe_3_O_4_–C1P1G1–XN nanocomposites possess a predominantly spherical morphology, with an average particle diameter of approximately 150 nm. Furthermore, the xanthohumol-loaded nanocomposite exhibited consistent d-spacing values, with the (1 1 1) and (2 2 0) planes indexed at 4.86 Å and 2.93 Å. The persistence of these diffraction signatures confirms that the incorporation of XN did not alter the crystalline structure of the magnetite nanoparticles. These results collectively demonstrate the successful formation of physically stable magnetite–polymer–drug architecture (Figure 11).

### 3.5. In Vitro Release Study

The in vitro release behavior of XN from the optimized Fe_3_O_4_–C1P1G1–XN nanocomposites was investigated to evaluate their suitability for sustained drug delivery. The casein–pullulan composite structure was designed to enhance the solubility of the encapsulated drug and to provide controlled release through diffusion and gradual matrix degradation.

**Figure 12:**
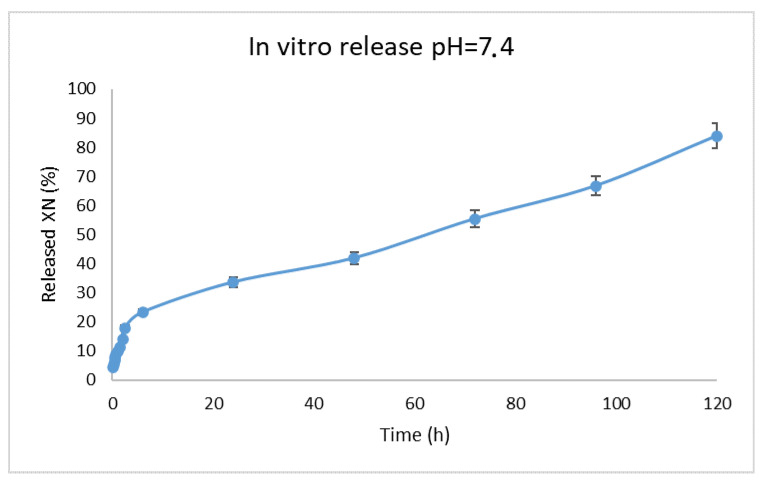
In vitro release profile of XN from Fe_3_O_4_-C1P1G1.0-XN nanocomposites.

The release profile demonstrated a slow and sustained release over a period of 120 h (Figure 12). During the initial 6 h, a moderate burst release was observed, corresponding to approximately 23% of the incorporated XN, which can be attributed to the desorption of drug molecules weakly associated with the particle surface. This was followed by a gradual release phase between 6 h and 48 h, reaching approximately 33% cumulative release. A more pronounced release occurred after 48 h, with cumulative values of 55.41 ± 1.06% at 72 h and 66.76 ± 0.60% at 96 h. At the end of the study (120 h), the cumulative release of XN reached 83.93 ± 1.10%.

The sustained release behavior indicates that the combination of casein and pullulan forms a slightly soluble, crosslinked composite matrix capable of effectively regulating XN diffusion. To elucidate the underlying release mechanism, the experimental data were fitted to various kinetic models, including zero-order, first-order, Higuchi, Korsmeyer–Peppas, and Hixon–Crowell models (Figure S1). Among them, the Korsmeyer–Peppas model showed the highest correlation coefficient (R^2^ = 0.9877), suggesting diffusion-controlled release kinetics. The calculated release exponent (n = 0.43) corresponds to Fickian diffusion in spherical systems [40]. These results indicate that XN release from the Fe_3_O_4_–C1P1G1–XN nanocomposites is governed primarily by molecular diffusion through the crosslinked casein network, with the pullulan coating acting as an additional diffusion barrier. The magnetic core serves as an inert structural component, contributing to mechanical stability without interfering with the release process [41].

## 4. Conclusions

This study demonstrates that the developed Fe_3_O_4_ casein–pullulan nanocomposites constitute a stable and adaptable platform for the delivery of hydrophobic bioactive compounds, exemplified by xanthohumol. The coating of magnetite nanoparticles with a hybrid protein–polysaccharide matrix significantly improved their physicochemical performance by reducing agglomeration and enhancing colloidal stability. Molecular docking and structural analyses confirmed that the casein–pullulan assembly is governed by extensive hydrogen bonding, resulting in a cohesive composite network capable of effectively encapsulating both the magnetic core and the drug payload.

The optimized formulation exhibited favorable morphology, preserved magnetic structure, and controlled drug diffusion through a biodegradable polymeric architecture. The in vitro release study revealed a sustained, diffusion-dominated release profile, supporting the ability of the casein–pullulan coating to prolong drug availability while maintaining structural integrity.

Overall, these findings establish casein–pullulan-coated Fe_3_O_4_ nanoparticles as a promising advanced drug delivery system. Future studies should focus on in vivo pharmacokinetics and biodistribution, functionalization for active targeting, and evaluation of additional therapeutic cargos, further advancing the potential of this platform in precision nanomedicine.

## Figures and Tables

**Table 1:** Variables in the 3^2^ experimental design.

Variables	Levels		
Independent			
X1: Casein Pullulan ratio	−1 (2:1)	0 (1:1)	+1 (1:2)
X2: Glutaraldehyde concentration	−1 (0.5%)	0 (1.0%)	+1 (1.5%)
Dependent			
Y1: Particle size (nm)			
Y2: Zeta potential (mV)			
Y3: DL (%)			
Y4: EE (%)			

**Table 2:** Composition of the obtained batches.

Batch	Levels	Casein (mg/100 mL)	Pullulan (mg/100 mL)	Glutaraldehyde (%)
Fe_3_O_4_-C2P1G0.5-XN	−1; −1	200	100	0.5
Fe_3_O_4_-C2P1G1.0-XN	−1; 0	200	100	1.0
Fe_3_O_4_-C2P1G1.5-XN	−1; +1	200	100	1.5
Fe_3_O_4_-C1P1G0.5-XN	0; −1	150	150	0.5
Fe_3_O_4_-C1P1G1.0-XN	0; 0	150	150	1.0
Fe_3_O_4_-C1P1G1.5-XN	0; +1	150	150	1.5
Fe_3_O_4_-C1P2G0.5-XN	+1, −1	100	200	0.5
Fe_3_O_4_-C1P2G1.0-XN	+1; 0	100	200	1.0
Fe_3_O_4_-C1P2G1.5-XN	+1; +1	100	200	1.5

**Table 3:** Results from the SEM-EDX elemental analysis.

	C1P1 G0.5 Fe_3_O_4_			C1P1 G1.0 Fe_3_O_4_			C1P1 G1.5 Fe_3_O_4_		
Measurement	C wt%	O wt%	Fe wt%	C wt%	O wt%	Fe wt%	C wt%	O wt%	Fe wt%
1	21.93	41.51	0.01	50.42	46.70	1.79	46.47	53.01	0.52
2	56.68	43.14	0.18	51.71	48.30	0.01	49.19	50.64	0.18
3	34.45	47.23	0.08	44.44	55.56	0.04	51.95	47.62	0.43
4	50.51	48.49	1.01	45.87	54.13	0.002	50.68	46.99	0.19
5	62.73	37.14	0.13	32.18	48.75	0.17	47.20	52.59	0.21
6	54.11	44.35	0.72	53.69	45.12	1.19	47.59	52.41	0.21
7	56.81	43.19	0.01	46.98	52.34	0.001	48.00	51.78	0.22
8	49.11	50.72	0.18	48.95	49.82	1.23	45.05	54.02	0.93
9	49.98	49.74	0.28	46.46	53.03	0.51	48.04	48.35	3.61
10	50.53	48.57	0.001	44.83	54.67	0.5	33.59	46.66	0.55
Av. ± SD wt%	48.68 ± 11.9	45.40 ± 4.2	0.26 ± 0.3	46.55 ± 5.8	50.84 ± 3.6	0.544 ± 0.6	46.77 ± 5.1	50.40 ± 2.7	0.70 ± 1.1

**Table 4:** Main characteristics of the obtained nine batches of nanoparticles.

Batch	Size (nm) ± SD	Zeta (mV) ± SD	DL (%) ± SD	EE (%) ± SD
Fe_3_O_4_-C2P1G0.5-XN	985 ± 11	−42.89 ± 1.4	7.4 ± 0.2	90 ± 0.9
Fe_3_O_4_-C2P1G1.0-XN	1164 ± 23	−37.86 ± 1.3	7.4 ± 0.4	88 ± 0.6
Fe_3_O_4_-C2P1G1.5-XN	1212 ± 102	−33.17 ± 2.8	6.3 ± 0.2	76 ± 0.8
Fe_3_O_4_-C1P1G0.5-XN	256 ± 19	−40.31 ± 1.2	6.4 ± 0.3	77 ± 0.9
Fe_3_O_4_-C1P1G1.0-XN	152 ± 9	−42.47 ± 1.2	5.7 ± 0.5	68 ± 0.8
Fe_3_O_4_-C1P1G1.5-XN	140 ± 8	−59.14 ± 1.2	5.4 ± 0.6	61 ± 0.5
Fe_3_O_4_-C1P2G0.5-XN	495 ± 42	−32.11 ± 1.1	7.5 ± 0.2	90 ± 1.3
Fe_3_O_4_-C1P2G1.0-XN	392 ± 33	−33.92 ± 2.1	7.2 ± 0.1	87 ± 1.4
Fe_3_O_4_-C1P2G1.5-XN	278 ±1 4	−35.51 ± 1.4	7.1 ± 0.3	85 ± 2.6

## Data Availability

The original contributions presented in this study are included in the article/Supplementary Materials. Further inquiries can be directed to the corresponding author.

## Supplementary Materials

The following supporting information can be downloaded at: https://www.mdpi.com/article/10.3390/polym18020256/s1, Table S1: Estimation of main effects and regression coefficients for the predictive mathematical models.; Figure S1: Graphical representation of the mathematical fitting of the release of XN from composite nanostructures to different kinetic models.; Figure S2: EDX spectra of Fe_3_O_4_ Cas/Pull nanocomposites crosslinked with 0.5/1.0/1.5% Glutaraldehyde.

## Abbreviations

The following abbreviations are used in this manuscript:

Cas/Pull	Casein–Pullulan
DL	Drug loading
DLS	Dynamic light scattering
EE	Entrapment efficiency
HP-β-CD	Hydroxypropyl-β-cyclodextrin
HP-β-CD/XN	Hydroxypropyl-β-cyclodextrin/Xanthohumol
MN	Magnetic nanoparticles
PBS	Phosphate-buffered saline
XN	Xanthohumol
