# Supplementary material for: Biopolymer Casein–Pullulan Coating of Fe3O4 Nanocomposites for Xanthohumol Encapsulation and Delivery
Source: Polymers (Basel). 2026 Jan 17;18(2):256. doi: 10.3390/polym18020256 (PMC12846283; doi:10.3390/polym18020256)
Supplement: Supplementary file 1 [file polymers-18-00256-s001.zip › polymers-4098656-supplementary.pdf]

Supplementary materials

Biopolymer Casein–Pullulan Coating of Fe_3O_4 Nanocomposites for Xanthohumol Encapsulation and Delivery

Table S1. Estimation of main effects and regression coefficients for the predictive mathematical models

Figure S1. Graphical representation of the mathematical fitting of the release of XN from composite nanostructures to different kinetic models.

Figure S2. EDX spectra of Fe_3O_4 Cas/Pull nanocomposites crosslinked with 0.5;1.0;1.5% Glutaraldehyde.

Table S2. Estimation of main effects and regression coefficients for the predictive mathematical models

		**Particle size**			
**Term**	**Coef**	**SE Coef**	**T-Value**	**P-Value**	**VIF**
Constant	564.50	9.54	59.15	0.001	
Cas:Pull					
2:1	602.3	13.5	44.63	0.001	1.33
1:1	-394.7	13.5	-29.25	0.001	1.33
GLD(%)					
0.5	-0.7	13.5	-0.05	0.958	1.33
1.0	-4.7	13.5	-0.35	0.730	1.33
Cas:Pull*GLD(%)					
2:1*0.5	-165.4	19.1	-8.67	0.001	1.78
2:1*1.0	19.9	19.1	1.05	0.310	1.78
1:1*0.5	59.3	19.1	3.11	0.006	1.78
1:1*1.0	-17.4	19.1	-0.91	0.374	1.78
		**Zeta Potential**			
**Term**	**Coef**	**SE Coef**	**T-Value**	**P-Value**	**VIF**
Constant	39.222	0.384	102.20	0.001	
Cas:Pull					
2:1	-1.698	0.543	-3.13	0.006	1.33
1:1	7.560	0.543	13.93	0.001	1.33
GLD(%)					
0.5	-1.724	0.543	-3.18	0.005	1.33
1.0	-2.513	0.543	-4.63	0.001	1.33
Cas:Pull*GLD(%)					
2:1*0.5	6.673	0.768	8.69	0.001	1.78
2:1*1.0	0.982	0.768	1.28	0.217	1.78
1:1*0.5	-6.338	0.768	-8.26	0.001	1.78
1:1*1.0	-3.436	0.768	-4.48	0.001	1.78
		**Drug Loading**			
**Term**	**Coef**	**SE Coef**	**T-Value**	**P-Value**	**VIF**
Constant	6.7319	0.0806	83.57	0.001	
Cas:Pull					
2:1	0.131	0.114	1.15	0.264	1.33
1:1	-0.447	0.114	-3.93	0.001	1.33
GLD(%)					
0.5	0.423	0.114	3.71	0.002	1.33
1.0	0.081	0.114	0.72	0.484	1.33

Cas/Pull*GLD(%)					
2:1*0.5	0.011	0.161	0.07	0.948	1.78
2:1*1.0	0.099	0.161	0.61	0.548	1.78
1:1*0.5	0.093	0.161	0.58	0.571	1.78
1:1*1.0	-0.026	0.161	-0.16	0.874	1.78

Encapsulation efficiency

Term	Coef	SE Coef	T-Value	P-Value	VIF
Constant	79.519	0.284	279.52	0.001	
Cas/Pull					
2:1	4.926	0.402	12.24	0.001	1.33
1:1	-11.074	0.402	-27.53	0.001	1.33
GLD(%)					
0.5	6.259	0.402	15.56	0.001	1.33
1.0	0.704	0.402	1.75	0.097	1.33
Cas/Pull*GLD(%)					
2:1*0.5	-0.704	0.569	-1.24	0.232	1.78
2:1*1.0	2.185	0.569	3.84	0.001	1.78
1:1*0.5	2.296	0.569	4.04	0.001	1.78
1:1*1.0	-1.148	0.569	-2.02	0.059	1.78

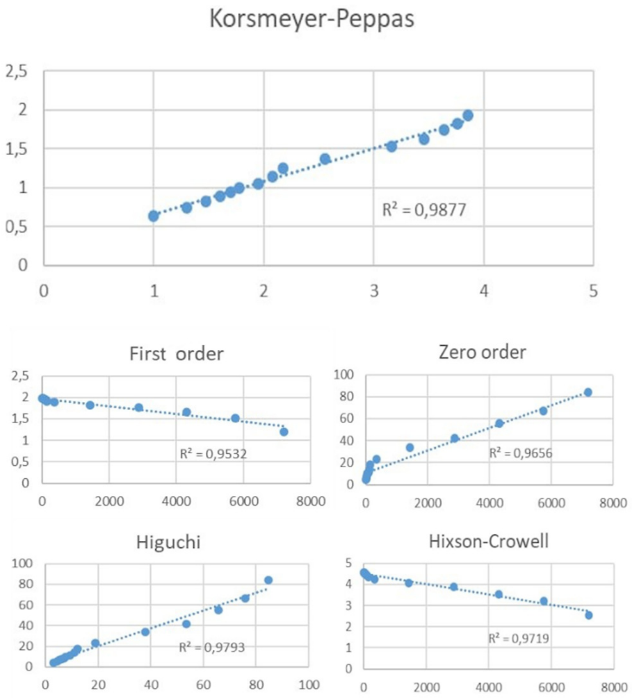

Figure S1. Graphical representation of the mathematical fitting of the release of XN from composite nanostructures to different kinetic models.

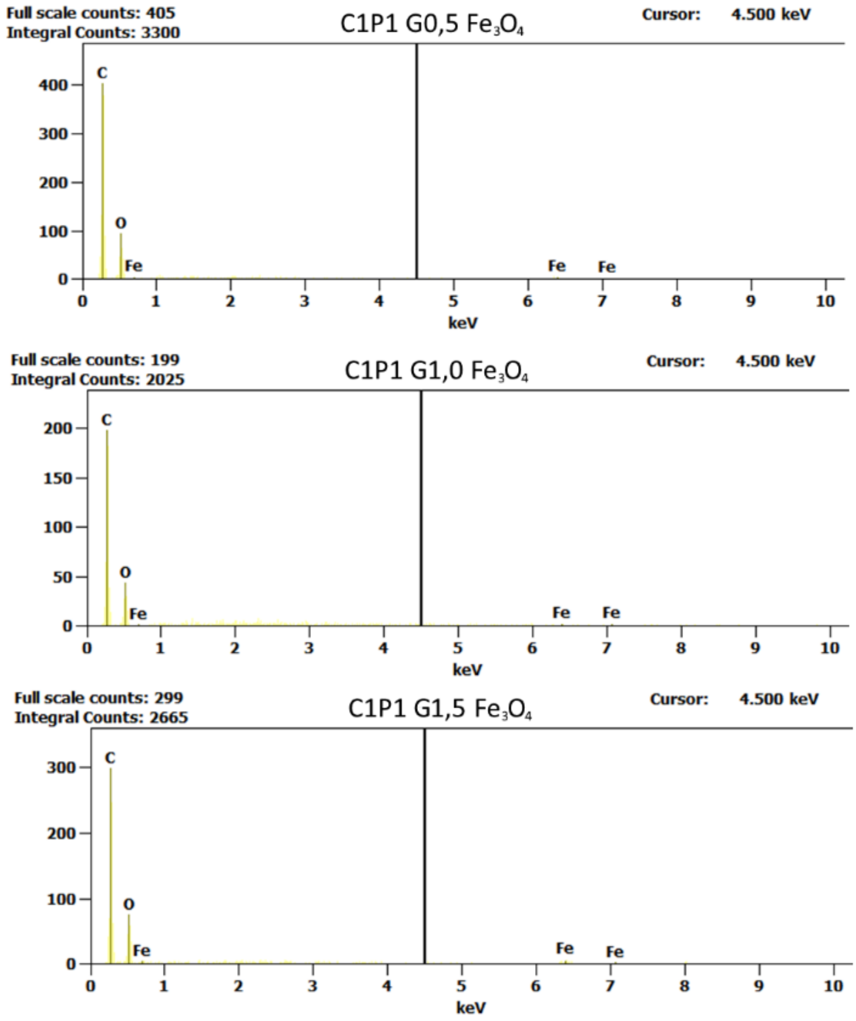

Figure S2. EDX spectra of Fe_3O_4 Cas/Pull nanocomposites crosslinked with 0.5;1.0;1.5% Glutaraldehyde.
